# Education—Treatment of Tooth-Pulp

**Published:** 1873-12

**Authors:** J. S. King


### EDUCATION—TREATMENT OF TOOTH-PULP.

BY DR. J. S. KING.

*Read before the Tennessee Dental Association.*

I appear in your midst almost an entire stranger. I have been kindly received by all the profession with whom I have formed an acquaintance, since my arrival in the State of Tennessee. These kind considerations and pleasant courtesies I fully appreciate, and I trust that my future relations with my professional brethren in this section of our common country may prove to be both pleasant and profitable to all parties. Since my arrival I have been solicited to present my name for membership in this Association. It has given me pleasure to do this, and I am much gratified to learn that I am accounted worthy of a place in your midst. In order that my professional brethren of this Association may know more of me, and more clearly understand the tone of my thoughts in relation to practical dentistry; that you may know the high respect and regard I entertain for my chosen profession, and also in compliance with requests from several members of this Association, I have decided to write a few statements,—and, perhaps, a thought or two,—and venture the trespass of a few moments upon your time and patience, in the perusal thereof in your hearing. If they prove to be crude and uninteresting, please censure not those through whose solicitations this paper was prepared.

In the winter of 1847—8, I had the honor to receive from the Ohio College of Dental Surgery a certificate of competency to practice the profession of dental surgery. In looking back over my professional history to that event, I confess to some feelings of humiliation. My after experience taught me how inadequately prepared I was for the discharge of the arduous duties I had taken upon me in the practice of my chosen profession. I do not propose to bring an accusation against my *alma mater*: her noble work in the past is well known to the profession, and, I trust, is but the prelude to her future achievements in the advancement of dental science and medico-dental education. I believe she has kept even pace with the improvements and attainments of this progressive age, and has run well the race with her compeers; and I trust her future career in the great work of dental education and in aiding in the elevation of professional honor and attainment, may ever be onward and upward.

In continuing my remarks before you, I will in my peculiar way proceed, first, to the consideration of the familiar and often discussed theme of professional education. In the mind of the thinking and intelligent dentist, it is of very great importance; and there is no one subject, when fully carried out and developed, which will so favorably affect the future triumphs of the profession. Much has been spoken and written on this interesting question, and its importance is ever before us for our consideration; and great is the work yet to be done for the elevation of the standard of dental education to that high position to which its importance entitles it to rise and occupy in the minds of all those who may aspire to a place and position in successful dentistry. The work to be done is for us to do. Those possessing a proper self-respect and a high respect for attainment in knowledge and skill, are, as far as their influence reaches, the custodians of the honor, the integrity and the triumphs of the profession; and in this capacity there is a negative duty devolving upon all, which should be faithfully discharged, without fear or favor, by every honorable operator; viz., an utter and entire refusal to aid or assist in any way in bringing into the profession the ignorant, the unqualified or the dishonorable.

Our positive duties are multifarious: duties to ourselves, which perhaps are not so often disregarded; duties to our patrons, in which the inward monitors of our hearts should ever be counseled with and heeded; duties to the profession, which should ever be active and on the alert to protect her honor and add to her attainments; and other duties there are too tedious to mention here, which should be squarely met and honestly discharged.

Our various dental schools and colleges throughout the country, for their better government, have adopted, each for itself, a specific standard of attainment in professional literature and dental science, which they profess to require and exact of each student in attendance, before they will confer upon said student the honors of the school. This is as it should be, is wise, and, when strictly enforced, will always be productive of great good to the profession. Let us sustain our dental schools as they should be sustained. Let us interest ourselves, and exact of every one of them a strict observance of these rules when giving out their certificate of qualification for dental practice. When our profession in one united phalanx adopts similar wise and prudential rules on this subject, then will our ranks be purged of empiricism and ignorance. Let us require of every dental student approaching any one in the profession for counsel or advice, to pledge himself to pursue to the end a thorough course of preparation, until he arrive at the full measure of professional knowledge and skill that will honor the instructor and the profession, an attainment which only can do justice to a confiding public, whom it is the province of the dentist to serve with the best skill of the age in which he lives.

Legislative enactments by the several states of the Union regulating the practice of dentistry within their borders, by the passage of wisely framed and equitable laws, is regarded by many good men in the profession as a thing much to be desired. It is thought that the public who patronize the profession would thereby be protected and greatly benefited, by preventing the unqualified man from the practice of the profession until he has obtained a fixed degree of progress in dental science, before he be allowed to openly offer his services to the people. Be this as it may, I, for one, would gladly welcome the day when a certificate of qualification from a properly authorized state board of examiners, or from some legally chartered dental college, would be the only conditions on which any one would be allowed to practice the profession of dental surgery within the boundaries of each and every state in this Union. Equitable legislation, no doubt, would greatly protect the public, and would do much to aid in dispelling the dark cloud of ignorance and charlatanism now overhanging the profession. But, as we are not now living under chartered privileges of this kind, let us arise from our lethargy, and do our duty in our present position; for there is much we can do in every time and place.

In our midst is a class of men difficult, perhaps, for us to to approach, over whom we should endeavor to exercise a correct and elevating influence. I admit, there are some who are only parasites, and all we can do is to shake them off, and turn from them in disgust, as unworthy of any professional consideration whatever. There are those, however, who are honest and honorable in all their motives and designs; whose life-work, as far as they know, is the practice of the same profession we ourselves are engaged in. The elevation of such in professional knowledge should not be overlooked by the more favored and gifted. Here is a work for our associations and societies to achieve. Let us draw these men into our various dental organizations throughout the country; let us inspire them with laudable ambition to strive for further improvement of themselves; let them, through the medium of a relationship with our various dental organizations, add to their limited stock of professional knowledge until they arrive at the full stature of professional accomplishment and success. When the light of our dental associations and societies radiates more fully and clearly in this direction, not only will these men become more educated and elevated, but all who through their influence and agency are brought into the profession will with them become more enlightened and accomplished in professional knowledge and skill. Let us, as men enlisted in one common calling, adopt for our future guidance that philanthropic motto, "The accomplishment of the greatest good for the greatest number."

In professional parlance and experience it is a well-demonstrated fact, that, when any one has attained to superlative excellence as an operator in dentistry, he cannot continue to labor for the same *fees*—per filling—that inferior operators are receiving. Here is a seeming vantage-ground, where ignorance, incompetency and retrogression take precedence, in the minds of many of our patrons, to competence, progression and skill. It is difficult for them to understand why I should charge $5.00 for a filling, and you charge $10.00. The unit—that is, the one filling—and the difference in the amount of money, crowd out of the mind all other ideas. The process of reasoning seems to be, that a filling is a filling, and therefore all fillings are the same and alike; or, that a dentist is a dentist, and therefore all dentists do about the same thing—except in charges. The true value of the objects to be obtained at in having teeth filled, or the ultimate success of the fillings they pay for, is lost sight of when accounts are to settle. Superiority in skill and success is thrown by them in the same heap with inferiority and failure, and, they will insist, must be paid for at the same rates. No! my brethren, this cannot be. A truly discriminating public will soon select from the common heap that which will permanently accomplish the object sought. And as superiority in skill involves great additional cost to the operator and additional time in the accomplishment of work, therefore it must be correspondingly remunerated, or, otherwise, abandon the field of professional effort. The intelligent and honest operator, having attained to a high degree of excellence in all his labors, cannot be induced to return by the way whence he came, to indulge in inferior operations for the poor privilege of bringing his labor down to the level of inferior *fees*. No! but rather let us increase our skill and progress in knowledge; let us go onward and upward in professional attainments and achievements unto perfection. And to those striving for higher improvement, let us communicate our knowledge, and inspire them with an honorable emulation, a knowledge and a zeal, that will honor both the man and the profession, in the accomplishment of many good and perfect works.

Before closing the perusal of this paper, please indulge me a little further, and allow me to call the attention of this Association to a single specialty in dental practice, which has attracted my attention during the past three years; viz., the uccessful treatment and preservation of the vitality of exposed dental pulps. This subject has been one of much interest, and has elicited mnch discussion during the score of years, and more, that I have been in the profession; and during these same years the brain and skill of many good operators have been exercised to know and do that, and that only, which would be most successful in such cases; and during these same years many thousands of teeth, that should have been saved, have been sacrificed by the profession, and their loss entailed upon suffering humanity, simply because the masses in the profession knew not how to do otherwise, in giving relief to their suffering patrons. The luster of a brighter day is now shedding its rays upon the profession. The forceps, in the majority of cases, must be laid aside for more sensible and humane treatment. All potent caustics and implements used for the destruction of living pulp-tissue of teeth must be abandoned. The agents and remedies for the preservation of the vitality and beauty of the teeth are in the possession of every honorable operator in the land, and it is only necessary that all in the profession understand their proper uses, in order to be almost universally successful in the preservation of the vitality of exposed tooth-pulps; of exposed and bleeding tooth-pulps; of exposed, bleeding and painful tooth-pulps: and if there are any other undesirable conditions associated with the exposed nerve-pulps of teeth, I include them also.

The system of treatment I now pursue in the above-described cases, differs materially in method from that which I had been pursuing for years previous to my subsequent discovery. True, I had been using the same remedies; but the principle and manner of application were different. Success with me then was not so certain, and the process was much more tedious, many cases requiring from two to five sittings before I would venture to cover the pulp, thereby consuming much valuable time. My first observations on my present mode of treatment were a surprise to myself. Not only was it a success in allaying all previous pain and suffering which many of my patients had endured, but the vitality of the pulp also proved to be successfully preserved. I now cover all exposed living pulps coming under my care for treatment at the first sitting of the patient, regardless of any previous painful condition; provided the pulp is not in the first stages of dissolution and sloughing. The following is my method of procedure;

First, I cut away the thin, weak borders of the cavity, as though I were at once to proceed to fill with gold; remove all the softened dentine I can from the cavity; and in doing this I many times unavoidably wound the pulp. I do not regard this wounding as being detrimental to the success of the subsequent treatment; yet I always endeavor to avoid wounding, if but for humanity's sake. At this juncture of proceedings, I usually apply rubber dam, and dry the cavity. If bleeding from wounded pulp continues, take a pellet of cotton, dip it in creosote, and apply to the bleeding surface until hemorrhage is thus arrested, and remove from the cavity all cuttings and clots. Being now ready for covering the pulp, take a glass tumbler, turn it bottom up, and mix thereon white oxide of zinc with wood creosote to the consistency of a soft jelly: this mixture I apply to the exposed pulp and *the surrounding sensitive dentine*, and, with a slight wrapping of cotton on the point of a small bur-headed instrument, gently press the mixture against the pulp, covering it perfectly but lightly, also keeping the mixture well away from the borders of the cavity. All thus far being done with care and precision, then proceed to mix, in the usual way, oxy-chloride of zinc to the consistency of cream, so that by its softness it will not displace covering of white oxide and creosote. With the second mixture, quickly fill the remainder of the cavity to the desired fulness. This completes the treatment of the pulp. The process is simple and easily executed: and if the pulp has not been painful prior to treatment, or has just been slightly exposed by recent cutting, I at once proceed to cut away enough of the oxy-chloride or outside covering to admit of the immediate insertion of a gold filling, thus completing the operation for the preservation of the tooth before allowing the patient to vacate the chair. But if, prior to the covering process, the nerve-pulp has been painful, or has been severely wounded, or very largely exposed, instead of at once finishing the operation, I dismiss the patient for some ten days or three weeks, with instructions to return at the expiration of said time, and allow me to finish my labors for the preservation of the tooth, by the insertion of a gold covering in the way and manner described above.

I have had a large experience in this mode of treatment since discovering its efficiency, over three years ago; therefore, I claim to know much in regard to the results thereof, all of which have been exceedingly gratifying to myself as well as many of my patrons, once suffering from severe and troublesome toothache. My purpose in calling the attention of this Association to this mode of treatment will not be fully realized until the profession has attained a success equal to my own, in the preservation of the vitality of the pulp and beauty of the teeth. Where the existence of a living exposed nerve-pulp invites to a trial of skill in its preservation, I will here take the position, that every individual in the exercise of his own choice has a right to claim and demand from the dentist whom he patronizes a complete success in the preservation of every case of exposed living nerve pulp: also, the time is at hand when he who uses any of the various potent caustics or agents, either mechanical or otherwise, (heretofore much used,) for the destruction of living tooth-pulp will be regarded as a relic of the darker days of the profession. Yea, more, I do here affirm, without fear of future controversion, that the dentist who hereafter continues the use of any drug or agent, by the use of which he destroys the living pulp-tissue of teeth committed to his care for treatment, will be guilty, not only of a crime against the profession, but also will be guilty of a crime against our patrons and our common humanity.

In regard to the mode or manner of action and effect upon the pulp-tissue by the agents used in this mode of treatment, or, rather, the rationale of their action producing these favorable results, and other questions kindred to these, I leave for future consideration and discussion.

